# Association among parents’ stress recovery experiences, parenting practices, and children’s behavioral problems: a cross-sectional study

**DOI:** 10.1186/s40359-025-02453-1

**Published:** 2025-02-15

**Authors:** Rikuya Hosokawa, Toshiki Katura

**Affiliations:** 1 Department of Human Health Sciences, Graduate School of Medicine, Kyoto University, 53 Kawahara-cho Shogo-in, Sakyo-ku, Kyoto, 606-8507 Japan; 2 Faculty of Nursing, Meiji University of Integrative Medicine, Kyoto, 629-0392 Japan

**Keywords:** Parental recovery, Stress, Parenting practices, Children’s behavioral problems

## Abstract

**Background:**

Parents can experience much stress from parenting, work, and household responsibilities. Parents’ stress recovery experiences, or their lack thereof, can affect parenting practices and influence children’s behavioral problems, which may thereby lead to difficulties for children later in life. Therefore, the relationships among these three factors deserve consideration. This study tested a model of the mediating role of parenting practices in the relationship between parents’ stress recovery experiences and children’s behavioral problems.

**Methods:**

Parents (*N* = 1,112) of 14-year-old children in the third year of junior high school in Japan completed a questionnaire, yielding 583 valid responses. To accurately determine the relationship among parents’ stress recovery experiences, parenting practices, and children’s behavioral problems, parents of children diagnosed with developmental disabilities and parents who did not respond to the required items in the questionnaire were excluded from the analysis. As a result, 536 of the 583 (89.0%) parents met the inclusion criteria. We conducted a path analysis, following the hypothesis that parents’ stress recovery experiences, via their parenting practices, are associated with children’s behavioral problems.

**Results:**

The path analysis results indicated that parents’ stress recovery experiences of relaxation and mastery were positively associated with positive nurturing attitudes, whereas mastery and control were negatively associated with negative nurturing attitudes. Furthermore, positive nurturing attitudes were negatively associated with externalizing and internalizing problem behaviors, whereas negative nurturing attitudes were positively associated with externalizing and internalizing problem behaviors. In other words, the hypothesis that parents’ stress recovery experiences of relaxation, mastery, and control reduce children’s behavioral problems via promoting nurturing parental attitudes was supported.

**Conclusions:**

The results indicate that the higher the level of parents’ stress recovery experiences, the lower the level of reported children’s behavioral problems. Parents’ stress recovery experiences correlated with parenting practices, which partially mediated the relationship of the parents’ stress recovery with children’s behavioral problems. The suggestion is that increasing parents’ stress recovery experiences, improving parenting practices and related behaviors, and strengthening the parent–child relationship are important measures that can be mutually beneficial for parents, children, and the overall family relationship.

## Background

In recent years, the traditional boundaries between work and personal life have increasingly blurred. Additionally, employees are experiencing major changes in their working conditions, resulting in increased stress levels [1]. In this context, communication technologies provide opportunities for employees to work outside the traditional office and beyond traditional working hours [2, 3]. The resulting changes require a better understanding of how employees spend their work and leisure time. Parenting stress has been widely recognized as a key factor influencing parenting behaviors and child outcomes, and parental coping strategies are critical in mitigating the adverse effects of stress. Coping strategies are defined as the cognitive and behavioral efforts individuals use to manage stressors and regulate emotional responses [4]. Research suggests that effective coping strategies, such as problem-solving and emotional regulation, can buffer the impact of stress on parenting practices and promote adaptive outcomes for children [5]. Conversely, maladaptive coping strategies, such as avoidance or rumination, may exacerbate stress and negatively affect parenting [6]. Therefore, this study focuses on examining the relationship between parental coping strategies and parenting practices to better understand how stress management can influence child development.

High parental stress is a significant environmental risk variable. It can increase parental depression [7] and marital conflict [8], negatively impact health [9, 10], decrease effective parenting [11, 12], and most importantly (in the context of this study), increase children’s behavioral problems [13]. Behavioral problems are among the most common health disorders in childhood. Children with persistent behavioral problems are at risk for poor health, social lives, and educational environments throughout their lives, causing distress to their families and leading to significant costs to society [14–16].

An important risk factor for behavioral problems is the type of parenting a child receives. Particularly, the home environment strongly influences children’s development of behavior-related abilities [17], and there is wide scientific recognition of parental stress having considerable impact on the home environment and being associated with rising behavioral problems in children. Indeed, parenthood can be difficult, and the daily demands of caring for and raising children pose a risk of stress for parents. As the typical primary caregivers, parents must meet the various physical and psychological needs of their children, including nutrition, protection, and care. In this context, the term “perceived stress” indicates responses to situations perceived as stressful, as well as a lack of resources to face those situations, and extensive research has documented increased stress among adults with children compared to childless adults. Then, in addition to the relationship of parental stress with the parenting burden and with the parental provision of resources to meet their children’s needs, parental stress affects parents’ psychological well-being, parenting practices, and the parent–child relationship. In fact, increased parental stress has been associated with negative parenting practices (e.g., corporal punishment) [18] and negative parent–child relationships [19]. It is unsurprising that recent decades have seen the academic community place substantial attention on the association between parental stress and children’s behavioral problems; numerous studies have suggested that the higher the level of self-reported parental stress, the more likely the children are to exhibit problem behaviors (both internalizing and externalizing). Given that parental stress negatively influences child development, it is important to investigate the relationship between parental stress and children’s behavioral problems.

As aforementioned, parenting can often be a challenging endeavor. It is also an ongoing and dynamic process, given that children’s needs develop and change as they grow [20]. Thus, parents must constantly adapt to their children’s changing needs. These needs influence the skills essential for parents to raise their children, including the ability to be emotionally involved in their children’s development. In addition to these needs, parents often have to adapt to changing social roles in the family system. When parents do not have the resources to adapt to these demands and changes, parental role-related stress can occur and is expressed in both psychological and physiological responses [21]. It is normal and almost inevitable for parents to experience some degree of parental stress as they adjust to changing demands and roles [22]. However, when parental stress persists unmitigated, it can have serious consequences for parents’ mental health, the parent–child relationship, and their children’s development [23]. Among parents with mental health disorders, such as depression and anxiety, parental stress can co-occur, and this factor is interrelated with other mental health factors. Subsequently, parental stress can affect child development, which can result in difficulties in children’s behavior, including behavioral problems at different stages of childhood [24].

As shown above, parental stress has widespread effects on parents and children and can affect the parent–child relationship [25]. Higher levels of parental stress increase parental depression, anxiety, and fatigue [26]. As a result, parents who report higher levels of parental stress also tend to have lower-quality parenting behaviors [27]. Parental stress is also associated with several adverse outcomes for children (e.g., increased emotional and behavioral problems, socioemotional dysfunction, and decreased social competence), either directly or indirectly through its effects on parents [28]. Thus, identifying modifiable mechanisms associated with parental stress recovery would be beneficial for both parents and children. In this study, we define parental stress recovery as the process of regaining emotional and psychological equilibrium after stress due to child-rearing responsibilities and other concurrent life demands, including work and household tasks [29, 30].

**Fig. 1:**
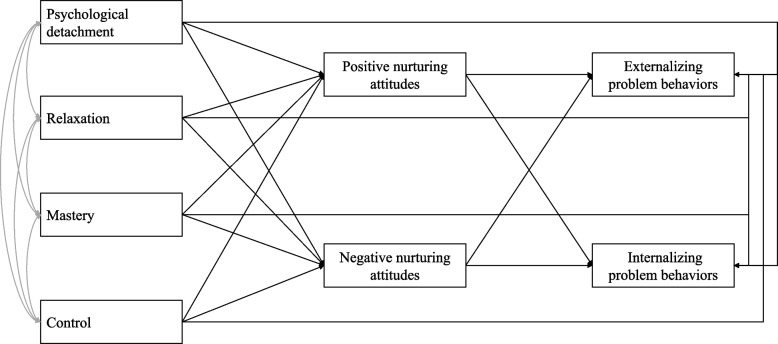
Hypothesized model

This study aimed to determine the relationship among parents’ stress recovery experiences, parenting practices, and children’s behavioral problems. We hypothesized that parents’ stress recovery experiences, via their parenting practices, are associated with their children’s behavioral problems (Fig. 1).

## Methods

### Participants

This study is part of a larger research project investigating the effects of child-rearing environment on children’s social development and adaptation. In this project, five-year-old children were recruited from 52 kindergartens and 78 nursery schools in Nagoya, Aichi Prefecture, a metropolitan area in Japan, in 2014. Since then, surveys have been conducted annually, with the current study using data collected in 2023. Accordingly, parents (*N* = 1,112) of 14-year-old children in the third year of junior high school answered a parent questionnaire, and 583 valid responses were obtained. To ensure that the data obtained would enable us to accurately determine the relationship among parents’ stress recovery experiences, parenting practices, and children’s behavioral problems, parents of children diagnosed with developmental disabilities and parents who did not respond to the required items in the questionnaire were excluded from the analysis. As a result, 536 of the 583 responses (89.0%) met the criteria.

### Ethics statement

Prior to data collection, parents were informed about the study objectives and procedures and that their participation in the baseline study was voluntary. Parents gave written informed consent for participation in the study on behalf of themselves and their children. Ethics approval for conducting this study was obtained from the Kyoto University Ethics Committee (E2322), and the study was conducted in accordance with the principles outlined in the Declaration of Helsinki.

### Measures

All key variables, including stress recovery, parenting practices, and child behavioral problems, were measured concurrently to capture their interrelated dynamics effectively [31].

#### Explanatory variable: parents’ stress recovery experiences

Data were measured using the Recovery Experience Questionnaire [32], which assesses what individuals do to restore psychological resources depleted by a stressful experience to their original level. This scale consists of 16 items divided into four subscales (psychological detachment, relaxation, control, and mastery). An example of a psychological detachment item is “I forget about work,” of a relaxation item is “I use time to relax,” of a control item is “I set my own schedule,” and of a mastery item is “I learn new things.” Respondents rate each item on a five-point Likert scale ranging from 1 (strongly disagree) to 5 (strongly agree) to indicate what they did over the weekend, and the scores for each item are summed to produce a total score. The Japanese version of the Recovery Experience Questionnaire has demonstrated good reliability and validity [33]. In this study, the subscales of the Recovery Experience Questionnaire exhibited acceptable to good reliability: psychological detachment (α = 0.77), relaxation (α = 0.81), control (α = 0.79), and mastery (α = 0.74). Scores for each subscale were summed separately to analyze the distinct dimensions of recovery experiences. Each subscale score under the stress recovery variable was analyzed independently without summing it into a composite score. This approach was employed to preserve the distinct theoretical constructs represented by each subscale, enabling a more granular analysis of their specific contributions to the outcomes of interest [34]. Scores were calculated by converting psychological detachment, relaxation, control, and mastery into *z*-scores.

#### Mediating variable: parenting practices

Parenting practices were measured using the Alabama Parenting Questionnaire [35–37], a self-reported parenting practices measurement tool frequently employed to assess the association of parenting with child outcomes. Its 42 items are scored on a five-point Likert scale that ranges from 1 (never) to 5 (always), and encompass five subscales, as described herein: inconsistent discipline (six items), corporal punishment (three items), inadequate supervision/control (10 items), positive parenting (six items), and engagement (10 items). Each subscale has demonstrated adequate internal consistency and construct validity. In this study, the subscales of negative nurturing attitudes (α = 0.72) and positive nurturing attitudes (α = 0.79) demonstrated sufficient internal consistency. A composite score for negative nurturing attitudes was calculated by converting the scores for the subscales of inadequate supervision/control, inconsistent discipline, and corporal punishment into a *z*-score, and averaging the *z*-scores. A composite score for positive nurturing attitudes was similarly calculated for each subscale of positive parenting and engagement, with higher scores indicating more positive nurturing attitudes.

#### Outcome variable: children’s behavioral problems

The Strengths and Difficulties Questionnaire [38] is a 25-item, validated assessment tool used to identify behavioral and emotional problems and prosocial behavior. In this study, the Japanese version was employed because of its high reliability and validity [39]. In this study, the Strengths and Difficulties Questionnaire subscales for externalizing problem behaviors (α = 0.71) and internalizing problem behaviors (α = 0.76) showed acceptable reliability. Specifically, participants responded to 20 items on behavioral and emotional problems, rated on a three-point Likert scale, across the following subscales: behavioral problems, hyperactivity/inattention, emotional symptoms, and peer relationship problems. Higher subscale scores indicated more severe emotional and behavioral problems. To calculate the total score for externalizing problem behaviors, the behavioral problems and hyperactivity/inattention subscales were converted into *z*-scores and averaged. Similarly, the total score for internalizing problem behaviors was calculated based on the scores for the subscales of emotional symptoms and peer relationship problems. Higher scores indicated more favorable parenting.

#### Demographic information

We collected self-reported demographic data on child sex, family structure (single parent or two parents), annual household income, and parental educational level (Table 1). To account for these factors in the analysis, we used sex and family structure as covariates (Tables 2 and 3).

**Table 1:** Participants’ characteristics

			**Externalizing problem behavior**			**Internalizing problem behavior**		
	***n***	***%***	***M***	***SD***	***p***	***M***	***SD***	***p***
**Child’s sex**								
Male	259	48.7	4.26	3.14	***	2.93	2.97	***
Female	273	51.3	3.15	2.56		4.06	3.39	
**Family structure**								
Single-parent family	39	7.3	4.69	3.62	*	4.71	4.53	*
Two-parent family	493	92.7	3.61	2.83		3.43	3.12	
**Annual household income (million JPY)**								
< 4	78	15.0	3.70	2.60		3.96	3.73	
≥ 4–8	281	54.1	3.78	2.94		3.61	3.16	
≥ 8	160	30.8	3.58	2.98		3.22	3.18	
**Maternal educational level**								
Middle or high school	83	15.70	3.52	2.77		3.91	3.42	
Junior college or vocational school	216	40.90	3.81	3.02		3.64	3.38	
University or graduate school	229	43.40	3.63	2.86		3.24	3.02	
**Paternal educational level**								
Middle or high school	107	20.6	3.91	2.85		3.88	3.79	
Junior college or vocational school	71	13.7	4.58	3.50		4.03	3.39	
University or graduate school	341	65.7	3.42	2.75		3.27	2.96	

^*^p < 0.05

^***^p < 0.001

**Table 2:** Correlations between parents’ stress recovery experiences, parenting practices, and children’s behavioral problems

	**1**	**2**	**3**	**4**	**5**	**6**	**7**	**8**	**9**	**10**	**11**	**12**
**Parents’ stress recovery experiences**												
1. Psychological detachment	-											
2. Relaxation	.610***	-										
3. Mastery	.132**	.349***	-									
4. Control	.325***	.564***	.279***	-								
**Parenting practices**												
5. Positive nurturing attitudes	.106*	.227***	.311***	.130**	-							
6. Negative nurturing attitudes	− .120**	− .152***	− .139**	− .192***	− .364***	-						
**Children’s behavioral problems**												
7. Externalizing problem behavior	− .090*	− .184***	− .098*	− .113*	− .294***	.472***	-					
8. Internalizing problem behavior	− .094*	− .120**	− .091*	− .106*	− .145**	.166***	.361***	-				
**Demographic information**												
9. Sex	− .030	.043	.046	.010	.160***	− .156***	− .191***	.174***	-			
10. Family structure	.007	.038	.036	− .035	.055	− .103*	− .094*	− .099*	.058	-		
11. Annual household income	− .014	− .001	.058	− .060	− .009	− .120**	− .019	− .075	.002	.169***	-	
12. Maternal educational level	− .030	.042	.139**	− .040	.033	− .062	.001	− .078	.011	.128**	.340***	-
13. Paternal educational level	.049	.091*	.070	− .009	.066	− .099*	− .082	− .087	.014	.097*	.300***	.428***

Sex: male = 1, female = 2. Family structure: single-parent family = 1, two-parent family = 2

^*^p < 0.05

^**^p < 0.01

^***^p < 0.001

**Table 3:** Unstandardized and standardized coefficients for the path analysis

**Construct**			***B***	***SE***	**β**	***p***
Psychological detachment	→	Positive nurturing attitudes	− 0.014	0.053	− 0.258	
Relaxation	→	Positive nurturing attitudes	0.165	0.063	2.619	**
Mastery	→	Positive nurturing attitudes	0.266	0.045	5.872	***
Control	→	Positive nurturing attitudes	− 0.029	0.051	− 0.574	
Psychological detachment	→	Negative nurturing attitudes	− 0.067	0.055	− 1.215	
Relaxation	→	Negative nurturing attitudes	0.005	0.065	0.072	
Mastery	→	Negative nurturing attitudes	− 0.094	0.047	− 2.000	*
Control	→	Negative nurturing attitudes	− 0.144	0.053	− 2.709	**
Positive nurturing attitudes	→	Externalizing problem behavior	− 0.142	0.042	− 3.410	***
Negative nurturing attitudes	→	Externalizing problem behavior	0.418	0.042	10.080	***
Positive nurturing attitudes	→	Internalizing problem behavior	− 0.097	0.047	− 2.073	*
Negative nurturing attitudes	→	Internalizing problem behavior	0.131	0.047	2.794	**

^*^p < 0.05

^**^p < 0.01

^***^p < 0.001

We assessed the correlations of parents’ stress recovery experiences (psychological detachment, relaxation, control, and mastery) with parenting practices (positive and negative nurturing attitudes), children’s behavioral problems (externalizing and internalizing problem behaviors), and demographic characteristics (child’s sex, family structure, annual household income, and parental educational level; Table 2). Path analysis was subsequently used to estimate the pathways between parents’ stress recovery experiences, parenting practices, and children’s behavioral problems.

To assess the model’s goodness of fit, we used the comparative fit index (CFI) [40], the incremental fit index (IFI) [41], and the root mean square error of approximation (RMSEA) [42]. A good model fit is indicated by CFI and IFI values above 0.90 and RMSEA values of 0.08 or less [43]. All statistical analyses were performed using SPSS version 29.0 and Amos version 29.0.

## Results

### Descriptive statistics

Data from 532 individuals who met the inclusion criteria were analyzed. Descriptive data are presented in Table 1. Our results showed that the child’s sex and family structure were significantly associated with behavioral problems. Therefore, and as mentioned in the Methods section, these variables were included as controls in the predictive model. The correlation results for the notated composite index of all study variables are presented in Table 2.

### Path analysis

**Fig. 2:**
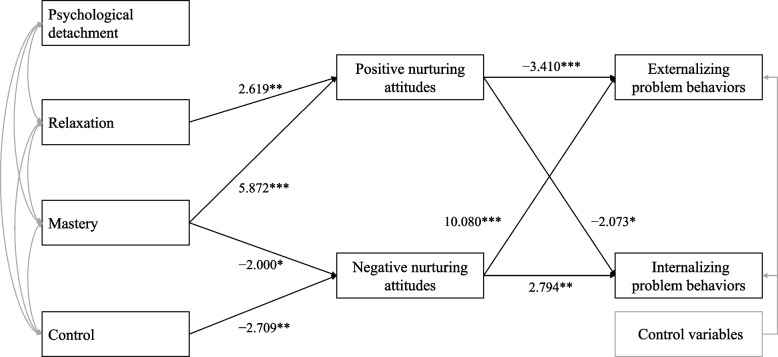
Statistically significant model. *Note:* This model includes the pathways that were statistically significant in the hypothesized model. Child’s sex and family structure were controlled for in the path analysis. Model fit statistics: χ.^2^ (8) = 17.20; CFI = 0.99; IFI = 0.99; RMSEA = 0.04. *p < 0.05; **p < 0.01; ***p < 0.001

In the hypothesized model, parents’ stress recovery experiences were used as the predictor variable, parenting practices as the mediating variable, and children’s behavioral problems as the outcome variable (Fig. 1). The results of the analysis are presented in Fig. 2. The findings showed that, among the subscales of parents’ stress recovery experience, psychological detachment was not associated with any of the input factors; relaxation was positively associated with positive nurturing attitudes; mastery was positively associated with positive nurturing attitudes and negatively associated with negative nurturing attitudes; and control was negatively associated with negative nurturing attitudes.

Second, positive nurturing attitudes were negatively associated with externalizing and internalizing problem behaviors (Fig. 2). Meanwhile, negative nurturing attitudes were positively associated with externalizing and internalizing problem behaviors. Thus, parents’ stress recovery experiences of relaxation, mastery, and control were associated with the suppression of externalizing and internalizing problem behaviors via positive or negative nurturing attitudes.

Parents’ stress recovery experiences were significantly associated with children’s behavioral problems through negative and positive nurturing attitudes (Fig. 2). This model fit the data well (goodness-of-fit statistics: χ^2^ (8) = 17.20, *p* < 0.001; CFI = 0.99; IFI = 0.99; RMSEA = 0.04), met the criteria for acceptable fit, and was excellent (Fig. 2).

## Discussion

Among parents’ stress recovery experiences, relaxation and mastery were positively associated with positive nurturing attitudes, whereas mastery and control were negatively associated with negative nurturing attitudes. Furthermore, positive nurturing attitudes were negatively associated with externalizing and internalizing problem behaviors, whereas negative nurturing attitudes were positively associated with externalizing and internalizing problem behaviors. In other words, the hypothesis that parents’ stress recovery experiences of relaxation, mastery, and recovery or control, through nurturing parental attitudes, reduce children’s behavioral problems was supported.

The findings of this study indicate that positive and negative nurturing attitudes significantly mediate the relationships between children’s behavioral problems and three dimensions of stress recovery—relaxation, mastery, and control. However, a mediating role of nurturing attitudes for the psychological dimension of detachment was not found. This suggests that the mechanisms linking psychological detachment to children’s behavioral outcomes may operate independently of nurturing attitudes. Previous studies have suggested that psychological detachment, as a cognitive and emotional disengagement process, may influence parenting indirectly through its impact on parental mental health or stress regulation [44]. This distinction emphasizes the need for future research to examine alternative pathways, such as parental self-regulation or emotional stability, that may mediate the effects of psychological detachment on child development.

In the present study, parents’ stress recovery experiences were positively related to their parenting attitudes. Parental stress is the experience of distress and discomfort resulting from the demands of the parental role and balancing work and family and is common among parents [45–47]. Parental stress has widespread effects on parents and children and can affect the parent–child relationship [48]. Higher levels of parental stress increase parental depression, anxiety, and fatigue. As a result, parents who report higher levels of parental stress also tend to have lower-quality parenting behaviors. Additionally, parental stress may be associated with several adverse child outcomes (e.g., increased emotional and behavioral problems, socioemotional dysfunction, and decreased social competence), either directly or indirectly, through its effects on parents.

The relationship between parental stress and children’s behavioral problems has been widely investigated; the evidence emphasizes parental stress as a predecessor of children’s behavioral outcomes and suggests that maladaptive child outcomes may be the result of parental stress [49]. Despite the bulk of knowledge on the matter, the potential dynamic interrelationships between parental stress and child outcomes remain underexplored. Moreover, parenting behaviors seem to be key for children’s behavioral development [50, 51], while parenting difficulties may result from parental stress, and higher levels of stress may lead to the application of more unhealthy parenting behaviors. These past findings imply the potential for the association of parental stress and children’s behavioral outcomes to be mediated by parenting behaviors. Specifically, parental stress, behaviors, and children’s behavioral outcomes seem to be interrelated, and parental stress may lead to suboptimal parenting, which may then result in children’s behavioral difficulties.

Children’s behavioral development is influenced by various parental behaviors, including, on the positive side, parental affection and encouragement, and, on the negative side, parental hostility and discouraging attitudes. Parental affection and encouragement refer to loving, caring, and responsive parenting, and low parental affection has been associated with children’s behavioral problems. With higher stress levels, parents may have difficulty regulating their emotions and, thereby, not be able to support their children emotionally. A potential consequence here would be stressed parents finding it more difficult to show affection and parental warmth and adopting more hostile attitudes toward their children. Decreased parental affection and increased hostility can lead to behavioral problems in children. Furthermore, having to deal with their children’s behavioral problems may cause parents to act less warmly and more hostilely toward their children, which are behaviors that can continue to exacerbate parental stress. Parental hostility, which includes parenting behaviors related to excessive control, negativeness, and antagonism toward their children, is associated with the development of children’s internalizing and externalizing problem behaviors and parental stress. These associations (i.e., parental hostility, children’s behavioral problems, and parental stress) have also been demonstrated to be reciprocal.

While parental stress impacts child development, another important factor—parenting practices—has also been widely reported to affect child development [52]. Particularly, previous studies have observed that parents high in parental stress are more likely to adopt an overly controlling approach to parenting. Authoritarian parenting practices have also been associated with both externalizing (e.g., aggression and hyperactivity) and internalizing behavioral problems (e.g., anxiety and social withdrawal) in children.

It is estimated that most parents experience some degree of anxiety regarding parenting, child behavior, and child development and may feel the need to seek professional advice and help. Thus, identifying modifiable factors associated with parental stress may be beneficial to health professionals who assist parents and children. Indeed, addressing parental stress is crucial today as families around the world face several stressors that impair their well-being.

Our findings emphasize the critical mediating role of parenting practices in the relationship between stress recovery and child outcomes. Although previous research has extensively documented the direct effects of parental stress on parenting behaviors and the subsequent impact of parenting on child outcomes, our study advances the understanding of this relationship by elucidating the full mediating pathway. Specifically, stress recovery may influence parents’ emotional availability, consistency, and responsiveness, which are pivotal in shaping children’s socioemotional development and behavioral regulation.

Parental stress recovery enhances self-regulation capacities, allowing parents to adopt more adaptive parenting practices. These improved practices, in turn, foster an environment conducive to positive child outcomes, including reduced internalizing and externalizing behaviors. For instance, stress recovery may enable parents to provide consistent discipline and emotionally supportive interactions, both of which are protective against the development of problem behaviors in children. This mediating pathway is supported by emerging evidence that links parental stress recovery to enhanced psychological flexibility and emotional availability, which are critical for effective parenting [53]. Furthermore, the importance of parenting as a mediator aligns with findings from other studies showing that parenting practices can mitigate or amplify the effects of parental stress on children’s developmental trajectories [54].

Moreover, our results suggest that child outcomes may benefit from interventions targeting parental stress recovery through improved parenting practices. This highlights the need for integrated approaches that address parental well-being as a means to enhance the parent–child relationship and, consequently, child developmental outcomes.

The relationship between stress recovery and psychological adjustment has been extensively discussed in the literature [55, 56], emphasizing the complex interplay between these constructs. This study focused on mediating effects, and future research should examine the main effects to clarify the extent to which the subscales of stress recovery exert direct influences on outcomes. Such analyses would contribute to a more nuanced understanding of these interactions.

Previous studies have detailed the impact of work–family conflict on parental stress, and our findings indicate the importance of effective stress recovery and coping strategies in mitigating these stress levels. Adaptive coping mechanisms, such as problem-solving and emotional regulation, play a crucial role in how parents manage and recover from stress [57, 58]. Future research should explore these coping strategies in greater depth to develop targeted interventions that support parental well-being.

The cross-sectional design of this study represents a significant limitation, particularly in the context of examining mediating relationships. Although the findings provide valuable insights into the investigated relationships, they do not allow for the determination of temporal order among variables. Mediation analysis inherently assumes directional processes, and this limitation prevents definitive causal inferences. Therefore, future research employing longitudinal or experimental designs is essential to confirm these findings and to further elucidate the underlying mechanisms.

Cross-sectional studies are useful for identifying associations; however, they are limited in their ability to disentangle complex processes such as mediation and causation. Recent literature underscores the importance of longitudinal designs in mediation research, as these provide stronger evidence for temporal and causal pathways [59, 60]. In this context, the findings of this study should be interpreted cautiously, and we strongly recommend that future research adopts longitudinal frameworks to confirm and expand upon these results.

This study has several limitations. First, it is a cross-sectional study, and causal inferences are not possible. Previous studies have found a bidirectional relationship between parental stress and children’s general behavior [61, 62]. Second, single bias is a risk because the variable of children’s behavioral problems was assessed by one person. Future research should include evaluations by teachers and children themselves. Third, our reliance on path analysis, rather than a full structural equation modeling framework, may limit the ability of the study to address measurement error and latent constructs, and we recommend that future investigations employ structural equation modeling to capture these complexities more comprehensively [63]. Finally, this study did not include an analysis of the main effects between each subscale of stress recovery and the outcome variables, leaving the extent of partial or full mediation undetermined. This represents a limitation of the current research. Future studies should incorporate data collection and analysis plans that explicitly examine these direct relationships. Addressing this issue would provide a more comprehensive understanding of the direct influences of the various aspects of stress recovery on psychological outcomes.

## Conclusions

Parenting is sometimes stressful, and parents’ recovery from stress can affect their parenting practices and their children’s behavioral problems, leading to potential difficulties for children later in life. Therefore, the relationships among these three factors deserve consideration. This study aimed to examine the direct relationships among parents’ stress recovery experiences, parenting practices, and children’s behavioral problems. Furthermore, it aimed to examine a model showing the mediating role of parenting practices in the relationship between parents’ stress recovery experiences and children’s behavioral problems.

Parents’ stress recovery experiences of relaxation, mastery, and control were positively associated with positive nurturing attitudes and negatively associated with negative nurturing attitudes. Furthermore, positive nurturing attitudes were negatively associated with externalizing and internalizing problem behaviors, whereas negative nurturing attitudes were positively associated with externalizing and internalizing problem behaviors. In other words, the hypothesis that parents’ stress recovery experiences of relaxation, mastery, and control reduce children’s behavioral problems through promoting nurturing parental attitudes was supported. Our findings suggest that strengthening the parent–child relationship by reducing parental stress and improving parenting practices is extremely important and mutually beneficial for both parents and children.

Furthermore, higher levels of parents’ stress recovery experiences were associated with lower levels of reported children’s behavioral problems. In addition, parental stress recovery was correlated with parenting style, and parenting style partially mediated the relationship between parental stress recovery experiences and children’s behavioral problems. It, therefore, seems critical to secure and design effective methodologies and initiatives to improve parents’ stress recovery experiences, parenting practices (along with related behaviors), and the parent–child relationship, as these betterments may be mutually beneficial for parents, children, and the family.

## Data Availability

No datasets were generated or analysed during the current study.

## Abbreviations

CFI: Comparative fit index
IFI: Incremental fit index
RMSEA: Root mean square error of approximation
